# Effect of Carbon Fibres on Electromagnetic-Interference-Shielding Properties of Geopolymer Composites

**DOI:** 10.3390/polym14183750

**Published:** 2022-09-08

**Authors:** Dimuthu Wanasinghe, Farhad Aslani, Guowei Ma

**Affiliations:** 1 Materials and Structures Innovation Group, School of Engineering, The University of Western Australia, Perth, WA 6009, Australia; 2 School of Engineering, Edith Cowan University, Joondalup, WA 6027, Australia

**Keywords:** geopolymer, electromagnetic shielding, carbon fibre, conductive composite

## Abstract

Many of the construction materials available are known to cause a drastic level of damage to the environment during their manufacturing stages. Hence, many researchers have attempted to formulate construction materials that are more environmentally friendly. Additionally, the rise in wireless communications in recent decades has seen a rapid increase in electromagnetic pollution and interference, which affects the functionality of sensitive electronic devices. This research is focused on fabricating a more sustainable construction material that could prevent electromagnetic interference for electronic devices housed inside. Carbon fibres of three different lengths were added in four variations to a geopolymer control mix to study their effect on electromagnetic interference shielding. The results showed that the amount of shielding produced by these composites increases with carbon fibre length and quantity. Morphological analyses showed that the interconnectivity of the fibres plays a crucial role in having a high level of shielding. While the flexural strength showed an improvement with the addition of carbon fibre, the compressive strength showed a slight reduction with the increase in carbon fibre length. The optimal level of shielding was produced by the specimen containing 0.7% of 12 mm carbon fibre, which was the maximum amount of fibre of any length used in this study; the optimal level of shielding generated was 43.43 dB within the frequency range of 30 MHz to 1.5 GHz.

## 1. Introduction

Cement and cement-based composites have been at the forefront of construction materials for decades due to their superior properties and ease of fabrication. However, with an increased emphasis on the environmental impacts of different sectors in engineering, research has been carried out to find construction materials that are more environmentally sustainable [1,2,3,4]. Geopolymer composite is one such material being developed as an alternative material to cement [5,6,7,8]. Geopolymer was first established in the 1970s as a thermosetting polymer with fire-retardant properties [9]. Since their introduction to engineering, geopolymers have undergone many alterations from subsequent research and have found applications in the construction industry. While geopolymers are environmentally friendly compared to existing construction materials, their fast setting time is one of the main reasons why they are yet to be used in the industry. Geopolymers have extremely low setting times, making it nearly impossible to use them in the construction industry [10,11]. The majority of the current research has focused on decreasing the setting time so that geopolymers can effectively be used in industrial applications [12,13,14,15,16].

Additionally, recent decades have seen a rapid increase in personal electronics, which have proven to make everyday lives easier. However, the radiation pollution caused by these devices has not been appropriately addressed. Electromagnetic (EM) radiation is known to cause other sensitive electronic devices to malfunction, cause health complications in humans, and used in espionage [17,18,19,20,21]. Traditionally, highly conductive metal sheets are used in building construction to prevent electromagnetic interference (EMI) caused this way. One of the main reasons metals creates good shields is that the high electrical conductivity of metals creates a Faraday’s cage when irradiated with EM radiation [22]. In the recent past, there has been an increased interest in developing a construction material that would act as a shield against EMI and would not require additional metal claddings. Much of this attention has been centred around cement and cement-based construction materials since cement is the most widely used construction material to date [23,24,25]. However, this research is focused on using geopolymers as EMI-shielding construction materials to overcome the negative environmental impacts of cement-based materials.

**Figure 1:**
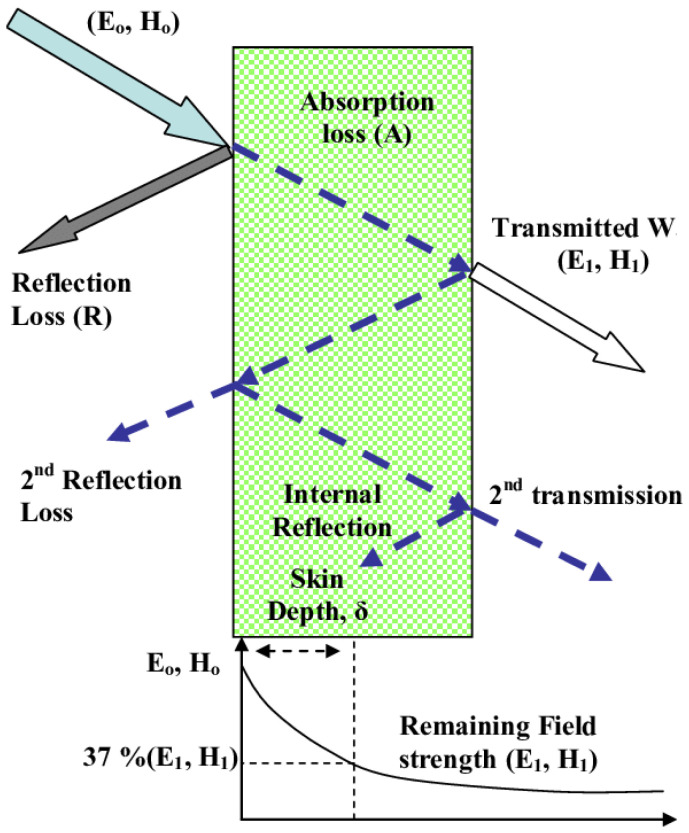
Interaction of EM waves with matter [28].

EMI shielding produced by a material can be divided into three different mechanisms known as reflection (*SE_R_*), absorption (*SE_A_*), and multiple reflection (*SE_M_*), as shown in Figure 1. Materials with high electrical conductivity, with mobile electrons and holes, are known to be good reflectors of EM waves [26]. The intensity of EM waves which manage to penetrate the material will undergo attenuation by the absorption mechanism. Absorption of EM waves mainly takes place when EM radiation interacts with the material, resulting in ohmic losses and the heating of the material [27]. The distance from the surface of the material where the intensity of the incident wave reduces to *e*^−1^ is known as the skin depth [23]. The third mechanism by which the EM radiation could lose intensity is multiple reflection, which occurs if the material consists of different surfaces or interfaces [26]. However, the multiple-reflection mechanism becomes negligible when the absorption is considerably large [27].

Several measurement techniques have been developed to measure the amount of EMI shielding produced by a material. These methods are mainly based on the frequency range of the EM waves. Measurement methods can be broadly categorised as open-field, transmission-line, shielded-room, and shielded-box techniques [27,29,30,31]. The EMI-shielding properties of the geopolymer specimens fabricated in this research were measured according to ASTM D4935-18, which is based on the transmission-line method [32]. This method is known to be a good way of measuring EMI-shielding properties of planar material and to have good repeatability [33].

The structure of geopolymers consists of a 3D network, which minimises the presence of free-moving electrons or holes [34,35,36]. The natal electrical conductivity of geopolymer is produced by the alkali ions present within the composites [37,38]. However, the mobility of these ions is not high, resulting in low electrical conductivity in geopolymers. One of the best ways to increase electrical conductivity would be to use a highly conductive additive, such as carbon fibre (CF).

CF is one of the most commonly used materials in the fabrication of conductive composites, especially for composites with insulating matrices, such as polymers [39]. Additionally, their low density and high tensile strength are also known to increase the overall tensile strength of the composite [40]. The use of CFs in composite fabrication has also increased recently due to lower manufacturing costs compared to several years earlier [41,42]. Prior research has used different types of CFs and shown to have better electrically conductive properties when CFs do not have a coating [43,44]. Hence, for this research, CFs that do not have a coating were used as the primary additive to impart electrically conductive and EMI-shielding properties. Previous research in using CFs for the fabrication of geopolymer composites has mainly looked at the mechanical, electrically conductive, self-sensing, fire-resistant, and morphological characteristics of these materials [45,46,47,48,49,50]. So far, there is no research that has investigated the possibility of using CF-reinforced geopolymer composites for EMI-shielding applications.

Since one of the objectives of this research is to fabricate an environmentally friendly construction material, the geopolymer mix that was established in prior research was used as the control mix to which the CFs were added in different percentages [51]. The control mix consists of fly ash and ground granulated blast-furnace slag (GGBFS), which are wastes generated in coal and steel industries, respectively, as binders [52,53,54]. Prior research on geopolymers has also shown that the addition of GGBFS can decrease the setting time [55].

## 2. Materials and Methods

Fly ash and GGBFS used in this research were procured by Cement Australia Pty Ltd. (Townsville, Australia) and Australian Steel Mill Services Pty Ltd. (Port Kembla, Australia), respectively. The chemical and physical properties of fly ash and GGBFS provided by the manufacturers are given in Table 1 and Table 2, respectively.

**Figure 2:**
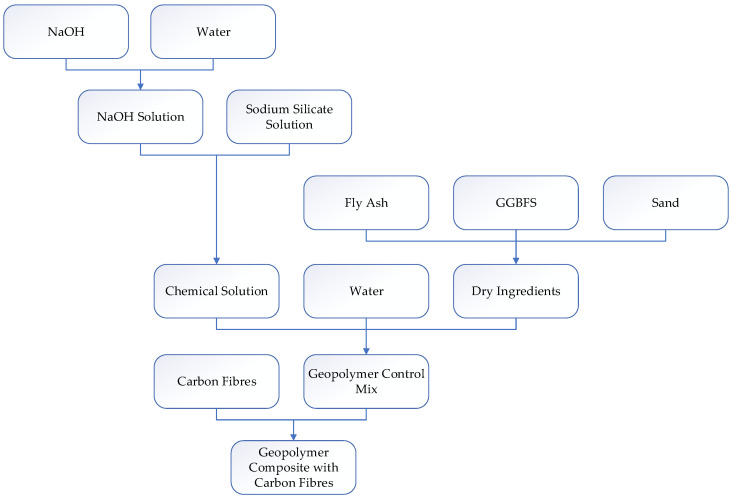
Flow chart of the fabrication process of the geopolymer composites.

The alkaline solution used consisted of 99% NaOH and sodium silicate, consisting of 28.7% SiO_2_, 3.2% Na_2_O, and 62.4% water. The solution was prepared by mixing the NaOH with water, followed by the sodium silicate solution. Prior to mixing with the binder, the alkaline solution was prepared by mixing the appropriate amount of chemicals and cooling it for 24 h. The mix design of the control mix is shown in Table 3. The amount of water indicated in Table 3 does not include the water content used to prepare sodium hydroxide solution. The complete process of fabrication of the composites is illustrated in the flow chart given in Figure 2.

CFs used to impart electrical conductivity and EMI shielding consisted of different aspect ratios. CFs with three different lengths were used in different weight fractions to assess the impact of their length and amount on all of the properties investigated in this research. The properties of the CFs used in this research are shown in Table 4. The CFs used in this research were unsized CFs since they have shown good electrically conductive and EMI-shielding properties in research conducted previously [56]. Compositions of the mixes containing CFs are shown in Table 5, where fibre fractions are given in weight percentages.

Fabricated specimens were tested for mechanical, electrically conductive, and EMI-shielding properties. For the assessment of compressive strength, cubes of the size of 50 mm × 50 mm × 50 mm were cast. The flexural strength of the mixes was assessed by casting and testing specimens with dimensions of 40 mm × 40 mm × 160 mm. For both compressive and flexural tests, three identical specimens were cast and tested per mix at a constant quasi-static test speed of 0.5 mm/min. Electrical resistivity was measured by using the four-probe technique using the Keithley 2100 multimeter. Specimens were dried at 110 °C for 24 h prior to testing to remove any remaining freestanding water. EMI shielding was measured using the Agilent E5071C vector network analyser and Electro-Metrics EM-2107A fixture in accordance with ASTM D4935-18 standard within a frequency range of 30 MHz to 1.5 GHz [32]. The thickness of all the specimens was made to be 10 mm to eliminate any discrepancies that would arise due to thickness variations. Similar to conductivity tests, specimens used for EMI shielding were also dried at 110 °C for 24 h to ensure that the reading would not be influenced by freestanding water. The distribution of CFs within the matrix was observed using the Zeiss 1555 VP-FESEM scanning electron microscope (SEM) after the specimens were coated with platinum.

## 3. Results and Discussion

**Figure 3:**
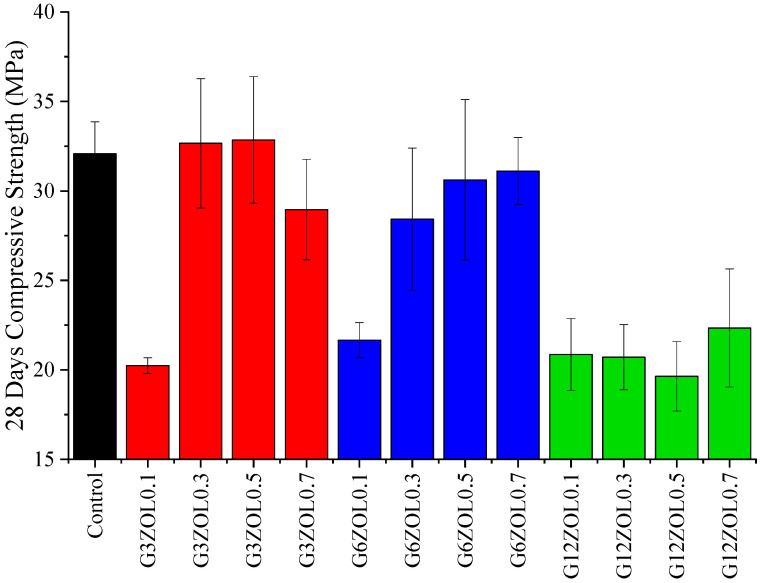
Compressive strength variation of the geopolymer mixes.

The compressive strength of the fabricated mixes was tested after 28 days, and their results are shown in Figure 3. On average, the addition of CFs to the geopolymer matrix has shown a detrimental effect on the compressive strength, with each mix showing a varying amount of reduction compared to the control mix. Only the mix containing 0.3% of 3 mm CFs has shown a compressive strength higher than the control mix. Mixes consisting of 3 mm CFs have shown an initial increase in the compressive strength followed by a gradual reduction, while mixes with 6 mm CFs have shown a gradual increase in the compressive strength followed by a slight drop in the last mix. Mixes containing 12 mm CFs have shown slight variation in their compressive strengths. Previous research has shown that the addition of CFs could lead to a higher porosity in geopolymer composites, which also reduces the compressive strength [57,58].

Apart from the porosity, another key aspect that could affect the compressive strength is the interface between the matrix and the additives [59,60]. The higher percentage of porosity created by the CFs can lead to some of these pores or air bubbles being trapped near the CFs and essentially creating a weaker interface between the matrix and the fibre. Additionally, these air entrapments could lead to a poor interface between the fine aggregates and the binder. During the fabrication process, it was observed that the addition of longer CFs led to a mix with higher porosity, and it was difficult to remove all of the air entrapped in the specimens due to the large fibre network. Similar phenomena of the drop in the compressive strength with the addition of CFs and fibres being pulled out have been reported in previous research [50,61]. Despite some mixes having low compressive strength, they have shown an adequate range of compressive strength to be used in industrial applications. 

**Figure 4:**
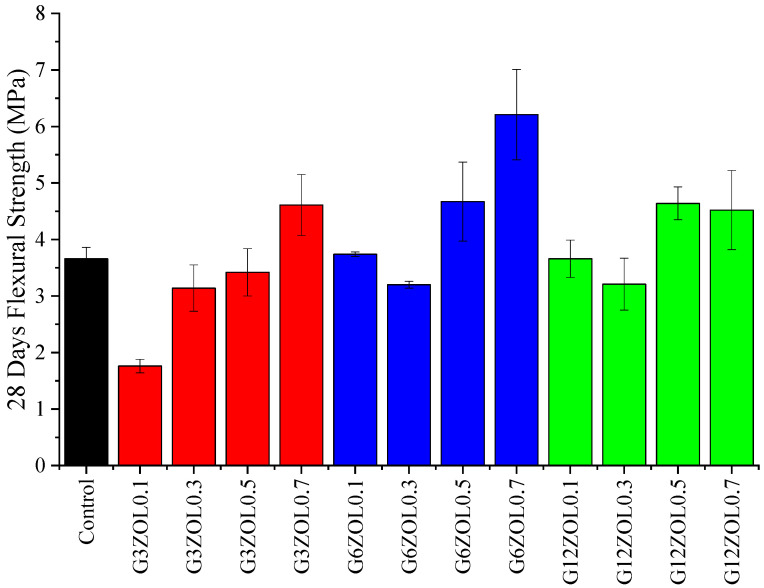
Flexural strength variation of the geopolymer mixes.

Variations in the flexural strength of the fabricated geopolymer specimens are shown in Figure 4. Due to the higher tensile strength of the fibres, the addition of fibres in composites is known to increase the overall flexural strength of the composites. However, when a small amount of CFs is added, it can be observed that the flexural performance of the specimens drops below the control mix. It is also known that the porosity of the specimens increases with the fibre addition, mainly due to the fibres trapping air within the composite [58]. While the specimens were vibrated using a vibrating table, it did not guarantee that all of the air entrapped within the composite would be removed during this process. For a given CF size, the flexural strength increases with the CF content. The highest flexural strength is shown by the specimen containing 0.7% of 6 mm CFs. Specimens containing 12 mm CFs do not show a significant variation in their flexural strength. However, these specimens also showed an increase in the flexural strength values when the CF content was increased. During the mixing process, it was observed that the length of the CFs has a profound effect on the workability, with the workability reducing with the fibre length. An increase in the length and the content of the fibre would lead to a more extensive network of fibres within the mix, leading to a higher amount of air entrapment. This would be the reason why these specimens do not show a significant increase in their flexural properties. Therefore, it can be assumed that further addition and increase in the length of the CFs added would not generate highly beneficial effects on flexural strength in these mixes. 

**Figure 5:**
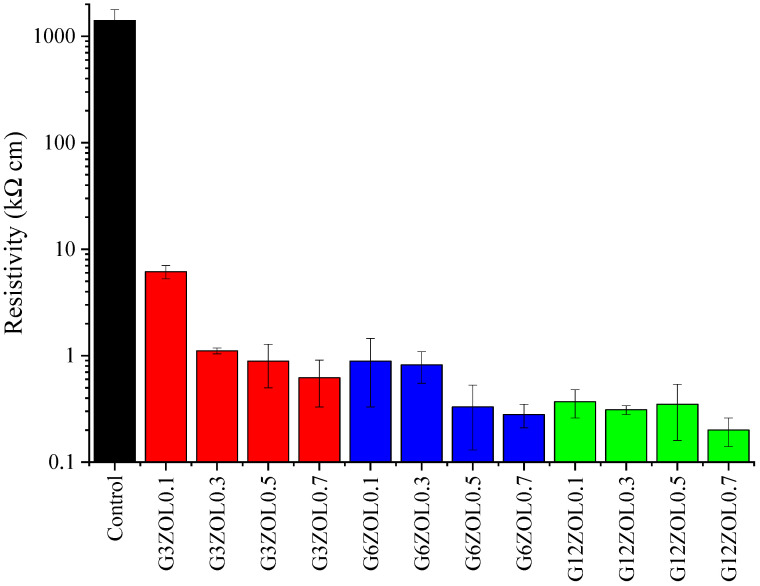
Resistivity variation of the geopolymer mixes.

The electrical conductivity of the geopolymer composite mixes has shown significant improvement with the addition of CFs, as shown in Figure 5. The average conductivity has shown an improvement with the size and the content of the CFs. One of the main reasons why the conductivity increases with the size of the CFs is due to the extension of the conductive network within the composite. Longer CFs would allow a higher percentage of fibres to overlap compared to shorter ones. Since the specimens were dried 24 h before the conductivity measurements, it can be assumed that the overall conductivity of the specimens is only due to the conductivity generated by the CFs and the inherent ionic conductivity of the geopolymer matrix. It can also be observed that the drop in resistivity decreases gradually with the CF content and size. From this observation, it can be assumed that further addition of CFs of any size would not produce a significant improvement in the electrical conductivity. The variation of the electrical conductivity of mixes containing 12 mm CFs is minimal, indicating that these specimens might have reached a saturation level of conductivity.

**Figure 6:**
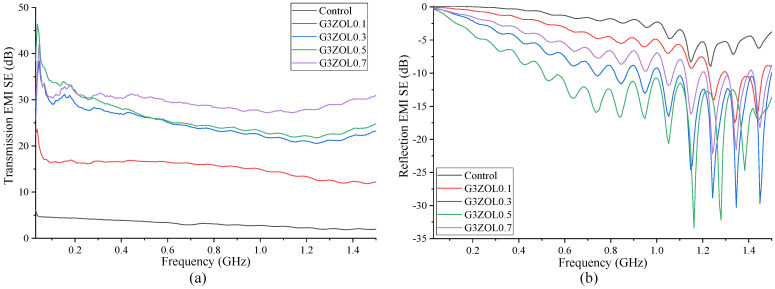
(**a**) Transmission EMI SE containing 3 mm CF; (**b**) reflection EMI SE containing 3 mm CF.

Both the reflection and transmission EMI-shielding properties of each mix were measured in accordance with ASTM D4935-18 standard [32]. Figure 6 shows the EMI-shielding properties of the specimens containing 3 mm CFs along with the control mix. Transmission-shielding properties show a gradual increase with the increase in the CF content. While the control mix shows an extremely low level of shielding, the addition of even 0.1% of CFs has a significant improvement on the shielding. However, when the CF content is increased further, the SE seems to reach an optimum level, as the increase in the SE does not show a significant variation. Additionally, mixes with higher CF percentages show near-identical shielding properties in the lower frequency range. However, with the increase in the frequency, the SE of the mixes also deviates in specimens with higher CF content having better SE. 

Reflection properties show an increase with the CF content up to 0.5% and a decrease with further addition. Generally, the reflection of EM waves from a material is known to take place when the material has a significant level of conductivity and can be influenced by the frequency of the EM waves as well [26,27]. Figure 5 shows that the electrical conductivity of the specimens containing 3 mm CFs varies considerably based on the CF content. However, the EM-wave reflection properties do not show a similar behaviour, indicating there could be several shielding mechanisms taking place in these specimens. One such mechanism that can take place is the multiple reflection that would arise due to porosity within the specimens, and these waves going out from the specimen would be similar to reflected waves. Specimens consisting of 0.5% of 3 mm CF also show a dip in the flexural strength, which could result due to an increase in the porosity within the specimens. 

**Figure 7:**
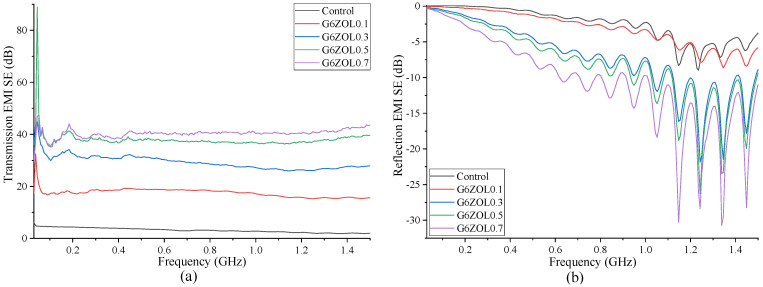
(**a**) Transmission EMI SE containing 6 mm CF; (**b**) reflection EMI SE containing 6 mm CF.

EMI-shielding properties of specimens containing 6 mm CFs are shown in Figure 7. Similar to specimens containing 3 mm CFs, these specimens also show an increase in their transmission- and reflection-shielding properties with the CF content. However, the level of increase in the transmission shielding shows a reduction with the increase in the CF content, indicating that the level of shielding will be reaching a saturation level. Both transmission- and reflection-shielding properties have shown the optimum level when the CF content is 0.7%. Comparison with the 3 mm CFs shows that 6 mm CFs have a slight improvement in the SE for the same CF content. 

**Figure 8:**
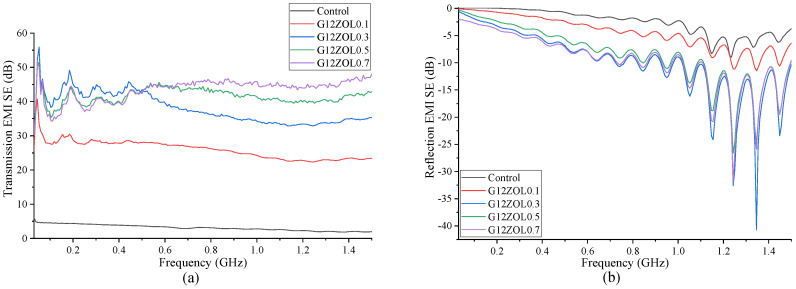
(**a**) Transmission EMI SE containing 12 mm CF; (**b**) reflection EMI SE containing 12 mm CF.

The 12 mm CFs have shown the best EMI-shielding properties out of all the mixes. The transmission- and reflection-shielding properties of these mixes are shown in Figure 8. Similar to other mixes, these also show that shielding properties reach a saturation level when the CF content is gradually increased. At lower frequencies, mixes with 0.5% and 0.7% of CFs show near-identical shielding properties. However, with the increase in the frequency, the mix with a higher amount of CFs shows better shielding characteristics. Reflection properties also show that the amount of EMI shielding produced by these specimens reaches a saturation level with the increase in the CF content.

Average EMI SE produced by the mixes with best shielding properties for the control mix and the mixes containing each CF type are 3.14 dB for the control mix, 29.58 dB for the mix with 0.7% of 3 mm CFs, 40.39 dB for the mix with 0.7% of 6 mm CFs, and 43.43 dB for the mix with 0.7% of 12 mm CFs.

## 4. SEM

**Figure 9:**
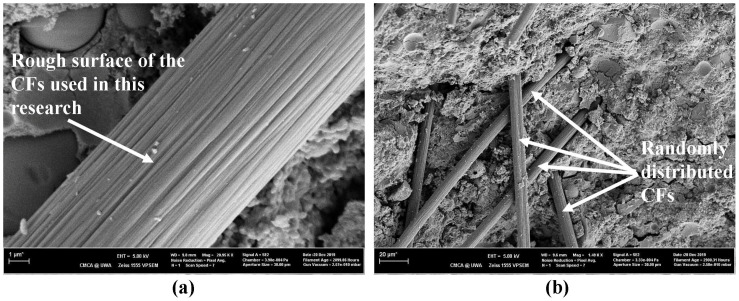
(**a**) Close up of CF used in the research; (**b**) distribution of CF in the geopolymer matrix.

SEM analysis was carried out to observe the distribution of the CFs within the geopolymer matrix. Figure 9a shows a close-up image of the CFs used in this research. It shows that the surface of the CFs consists of grooves, which would help them bond to the matrix. Figure 9b shows how the CFs have been distributed within the geopolymer matrix. Images showed that the CFs within these specimens were distributed randomly and overlapped with each other. Since a good conductive network is a crucial factor in having high EMI-shielding properties, such overlapping is a critical requirement to obtain the properties sought in this research. In addition to showing the distribution of CFs, these images also revealed the presence of other constituents of the geopolymer. SEM analysis further correlated that higher loadings of CFs lead to more interconnectivity, which would result in higher EMI SE. Additionally, SEM images also showed that longer CFs are better at creating the extensive conductive network necessary for higher SE. Some of the SEM images showed that the CFs have been pulled out from the matrix when the specimen was broken, indicating the interface weakness between the fibre and the matrix. However, such observations were not widespread throughout the specimens and can be due to air getting trapped at the interface between the fibre and the matrix. A small amount of agglomerations of the CFs were also present when the CF content in the specimen was at a high percentage. This kind of clustering was unavoidable during the fabrication process, even though they were mixed with water to ensure that they were separated and mixed with the matrix as much as possible. However, the EMI-shielding and electric-conductivity results did not show any variation due to such clustering. One of the key challenges of SEM analysis was that, since the matrix material is electrically insulating, the specimens needed to be coated with platinum for the SEM analysis. However, even this could not prevent the charging of the specimen that takes place due to the buildup of charges on the surface that results in poor quality images. Hence, for some specimens, several attempts had to be made before a high-quality image could be obtained. 

## 5. Conclusions

This research was focused on investigating the possibility of using geopolymer composites in EMI-shielding applications. In order to improve the EMI-shielding properties of these composites, CFs with different aspect ratios were added and tested for a variety of properties. Based on the results obtained, the following conclusions could be drawn:

1. Twenty-eight days’ compressive strength showed an increase with the increase in the CF content for each CF size. However, on average, the compressive strength showed a drop with the CF size.
2. The flexural strength showed gradual improvement with the increase in the CF content for a given size. It could be observed that, in order to increase the flexural strength of these composites significantly, the CF content needs to be higher than 0.5%.
3. The electrical conductivity increased with the size and the content of the CFs. However, when the CF of a given size increased, the rate of increasing the conductivity gradually dropped, indicating that the electrical conductivity would reach a saturation level.
4. Increasing the CF content showed a gradual increase in the EMI shielding. However, the rate of increase in EMI shielding was reduced with increasing CF content, indicating saturation of EMI-shielding properties. Increasing the CF size also showed a beneficial effect on the shielding properties, with 0.7% of 12 mm CFs showing the best shielding properties, which is 43.43 dB over the tested frequency range.
5. SEM analyses of the composites showed that the CFs have been distributed evenly throughout the matrix in random orientations mixed with other constituents.

## Figures and Tables

**Table 1:** Chemical and physical properties of fly ash.

Chemical Properties		Physical Properties	
CaO	3.30%	Relative density	2.29
SiO_2_	50.40%	Moisture	<0.1%
Al_2_O_3_	31.50%	Relative water requirement	93%
Fe_2_O_3_	10.40%	Sulphuric anhydride	0.10%
SO_3_	0.10%	Chloride ion	0.00%
MgO	1.10%	Chemical composition	92.30%
Na_2_O	0.30%	Loss on ignition	1.10%
K_2_O	0.50%	Strength index	102%
SrO	<0.1%		
TiO_2_	1.90%		
P_2_O_5_	0.50%		
Mn_2_O_3_	0.20%		
Total alkali	0.60%		

**Table 2:** Chemical and physical properties of GGBFS.

Chemical Properties		Physical Properties	
FeO	1.30%	Bulk density	850 kg/m^3^
CaO	38–43%	Glass content	>85%
SiO_2_	32–37%	Angle of repose	Approx. 35°
Al_2_O_3_	13–16%	Chloride ion	<0.025%
MgO	5–8%		
TiO_2_	1.50%		
MnO	0.50%		
Hydraulic index	1.7–1.9%		

**Table 3:** Composition of the control mix.

Mix	Label	Fly Ash	GGBFS	Water	45/50 Sand	NaOH	Sodium Silicate
Control	GC	0.56	0.44	0.17	0.81	0.11	0.29

**Table 4:** Properties of the CF.

Type and Length of CFs	Tensile Strength (MPa)	Tensile Modulus (GPa)	Electrical Resistivity (Ω∙cm)	Density (g/cm^3^)	Fibre Diameter (µm)	Carbon Content (%)
Unsized 3 mm	4137	242	1.55 × 10^−3^	1.8	7	95
Unsized 6 mm	4137	242	1.55 × 10^−3^	1.8	7	95
Unsized 12 mm	4137	242	1.55 × 10^−3^	1.8	7	95

**Table 5:** Composition of the mixes containing CF.

Mix Label	CF Length	CF Content	Total Alkaline Solution
G3ZOL0.1	3 mm	0.1%	0.4
G3ZOL0.3		0.3%	0.4
G3ZOL0.5		0.5%	0.4
G3ZOL0.7		0.7%	0.4
G6ZOL0.1	6 mm	0.1%	0.4
G6ZOL0.3		0.3%	0.4
G6ZOL0.5		0.5%	0.4
G6ZOL0.7		0.7%	0.4
G12ZOL0.1	12 mm	0.1%	0.4
G12ZOL0.3		0.3%	0.4
G12ZOL0.5		0.5%	0.4
G12ZOL0.7		0.7%	0.4

## Data Availability

Data will be made available on request.
